# Circular RNA and tumor microenvironment

**DOI:** 10.1186/s12935-020-01301-z

**Published:** 2020-06-03

**Authors:** Huixin Song, Qiaofei Liu, Quan Liao

**Affiliations:** Department of General Surgery, Peking Union Medical College Hospital, Chinese Academy of Medical Science & Peking Union Medical College, Beijing, 100730 China

**Keywords:** CircRNA, Tumor microenvironment, Immunotherapy

## Abstract

Circular RNAs (circRNAs) are small non-coding RNAs with a unique ring structure and play important roles as gene regulators. Disturbed expressions of circRNAs is closely related to varieties of pathological processes. The roles of circRNAs in cancers have gained increasing concerns. The communications between the cancer cells and tumor microenvironment (TME) play complicated roles to affect the malignant behaviors of cancers, which potentially present new therapeutic targets. Herein, we reviewed the roles of circRNAs in the TME.

## Introduction

CircRNAs are a class of highly abundant endogenous RNAs with a unique covalently closed, single-stranded, complete ring structure with no free 3′ or 5′ ends. CircRNAs are highly abundant and stable in the cytoplasm, as most of them are resistant to RNase. A variety of gene structures generate circRNAs mainly via a type of alternative RNA splicing called ‘back-splicing’ [1], regulated by canonical splice signals [2]. In 1976, Sanger et al. first investigated the existence of circRNA in viroid [3]. In the following decades, various circRNAs were identified in human cells, although for a long time they were thought to be just junk byproducts of RNA splicing errors [1]. It was not until novel sequencing technologies made the transcriptional profile of the entire human genome available did these molecules gain attention. The vital roles of circRNAs have increasingly come into light with the development of RNA sequencing technology and numerous efficient specific algorithms to detect and quantify genome-wide circRNA expression from RNA sequencing data [4].

**Fig. 1:**
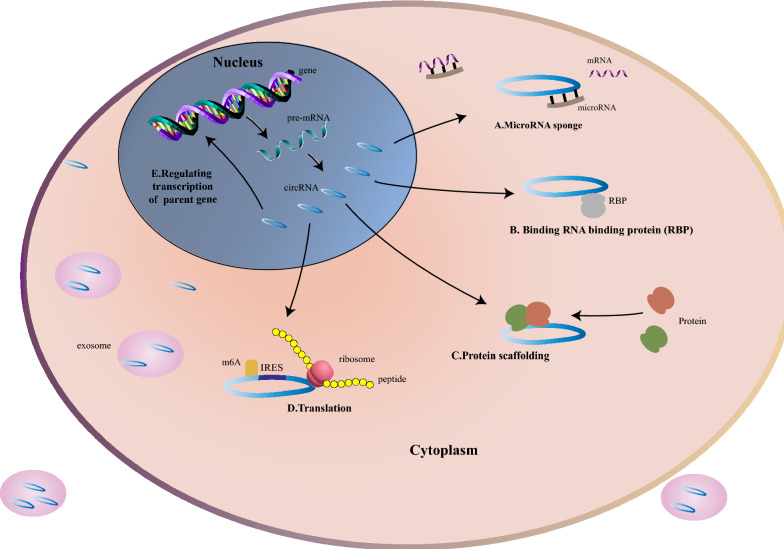
Molecular mechanism of circRNAs. **A** circRNAs sponge miRNA to release mRNA and regulate transcription indirectly; **B** circRNAs with RNA-binding protein-binding motifs sponge or decoy RNA binding proteins (RBPs) to regulate transcription; **C** circRNAs serve as a scaffold for proteins; **D** circRNAs with internal ribosome entry sites (IRESs) can be translated into peptides or proteins, driven by m6A; **E** circRNAs regulate transcription of parental genes

The special characteristics of circRNAs, such as extensive distribution, stability, and cell type-specific and tissue-specific expression, ultimately shape their functional roles, such as cell cycle regulation, proliferation, and apoptosis [5, 6]. Aberrantly expressed circRNAs exert tumor-suppressive or oncogenic functions by microRNA(miRNA) sponge function, posttranscriptional regulation [7, 8], circRNA-derived pseudogene translation [9, 10] and interaction with protein (Fig. 1), thereby affecting cancer initiation, development, metastasis [11–20] and therapy resistance [21–25]. These molecules have already become a novel area of interest and promising molecular focus in the diagnosis and treatment of cancer.

**Fig. 2:**
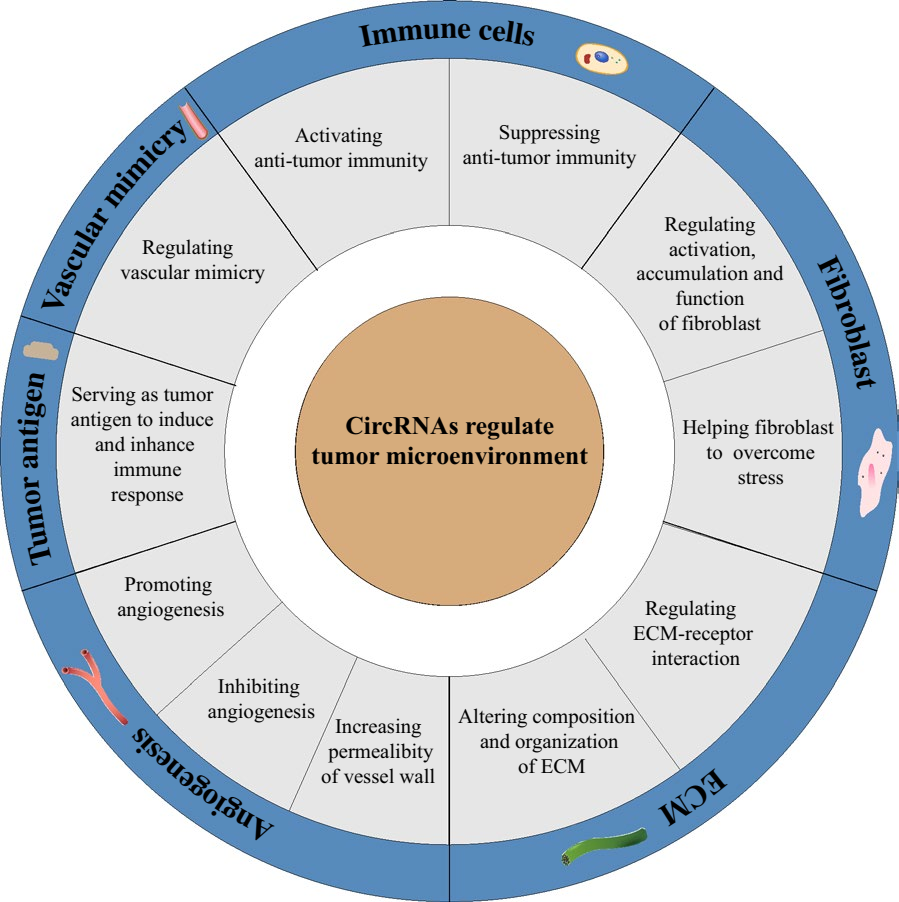
General description of the roles of circRNAs in TME. CircRNAs modulate TME through several different aspects: immune cells, fibroblasts, ECM, angiogenesis, vascular mimicry and serving as tumor antigens

TME, nourished by the vasculature, consists of the cell compartment (which includes tumor cells, immune cells, and other nonmalignant cells), extracellular matrix (ECM) and abundant signaling molecules. It is the indispensable ‘soil’ for in situ and metastatic tumor cell growth. The intimate, complicated and rapidly changing crosstalk between cancer cells and the surrounding structure exerts a significant influence on tumor initiation, development and therapeutic response [26–32]. Evidence also reveals that cancer-related inflammation in the TME is an essential hallmark [33]. The TME can both improve and inhibit therapeutic efficacy and may have variable activation status. Modifying or regulating specific factors or cells in the TME is particularly beneficial to treat tumors, one example being checkpoint inhibitors, which have already made great strides in cancer treatment in the past decade [26–30, 33–37]. The mechanisms of biogenesis of circRNA and its roles to directly regulate the malignant behaviors of cancer cells have been widely reviewed before, so it is beyond the scope of this review [38–41]. In this review, we focused on the roles of circRNAs in the TME and discuss the potential application of circRNAs in tumor therapy (Fig. 2), to provide novel ideas and methods to uncover a rational design for combinational therapies to overcome therapeutic resistance.

## CircRNAs play roles in TME regulation

### CircRNA and immune cells

**Fig. 3:**
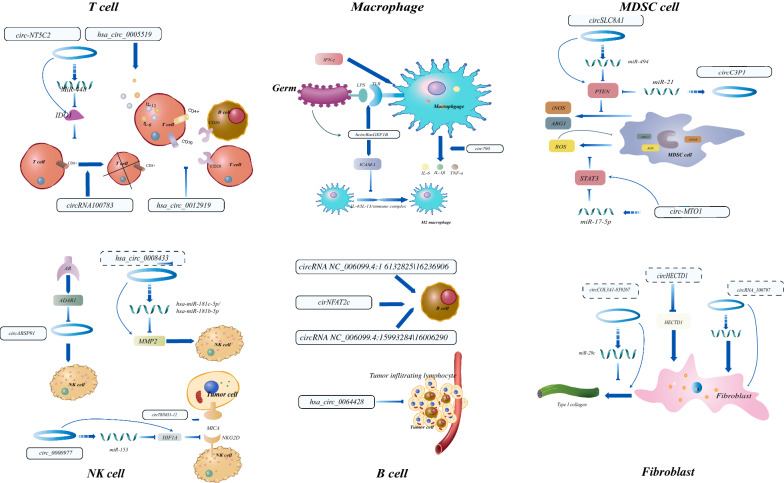
Functions and mechanisms of circRNAs to modulate varieties of immune cells. Specific circRNAs and their targets were presented

Tumor cells and different types of immune cells influence each other at various stages of cancer, which contributes to the complexity of the TME. Immune cells can be remodeled to favor tumor cell proliferation, evasion and metastasis. Among immune cells, antigen-presenting cells (including dendritic cells (DCs), epithelial cells and B cells), natural killer cells (NKs) and lymphocytes (especially T cells) are crucial for tumor suppression, while tumor-associated macrophages (TAMs), regulatory T cells (Tregs) and myeloid-derived suppressor cells (MDSCs) are considered to play immunosuppressive roles. The previously reported studies about the relationships between circRNAs and immune cells are summarized in Fig. 3 and Table 1.

**Table 1:** Representative circular RNAs targeting immune cells

CircRNA	Immune cell	Expression	Targeted miRNA	Downstream target	Cancer type	Immune-related functions	Clinical correlation	Reference
circ-LAMP1	T cells	Up-regulated	MiR-615-5p	DDR2	T-cell lymphoblastic lymphoma	Inhibiting cell apoptosis in T-LBL cells	/	[49]
circ-NT5C2	T cells	Up-regulated	MiR-448	IDO1	Osteosarcoma	Enhancing the CD8+ T cell response	/	[45]
hsa_circ_0005519	T cells	/	Hsa-let-7a-5p	IL-13 and IL-6	/	Activating T cell related immune response	/	[42]
hsa_circ_0012919	T cells	/	MiR-125a-3p	/	/	Mediating activation, proliferation and differentiation of T cells and B cells and immune response	/	[43]
circRNA100783	T cells	/	/	/	/	MediatingCD28-related CD8(+) T cell ageing and global immune senescence	/	[47]
hsa_circ_0064428	B cells	Down-regulated in patients with high tumor-infiltrating lymphocytes	Hsa-miR-7977, hsa-miR-4530, hsa-miR-4692, hsa-miR-4514 and hsa-miR-4645	/	Hepatocellular carcinoma	Upregulating TIL frequency	Tumor size, metastasis, overall survival	[61]
circ_0000977	NKs	Up-regulated	MiR-153	HI1FA, ADAM10	Pancreatic cancer	Inhibiting NK cell lysis, resulting immune escape of pancreatic cancer cells	/	[69]
circARSP91	NKs	Down-regulated	/	/	Hepatocellular carcinoma	Strengthening the cytotoxicity of NK cells, enhancing innate immune surveillance	Overall survival	[65]
circTRIM33–12	NKs	Down-regulated	MiR-191	TET1,5hmC	Hepatocellular carcinoma	Enhancing immune response, preventing cancer progression	Overall survival	[66]
hsa_circ_0008433	NKs	/	Hsa-miR-181c-5p and hsa-miR-181b-5p	MMP2	/	Inducing NK cells to attack arterial elastic fibers and remodel vessels	/	[63, 64]
circ790	Macrophages	Down-regulated	/	IL-6, IL-1β and TNF-α	/	Modulating the secretion of specific cytokines and mediates immune response	/	[92]
hcircRasGEF1B	Macrophages	Down-regulated	/	ICAM-1	/	Promoting of LPS response and regulating macrophage polarization	/	[93]
circHECTD1	Macrophages	/	/	/	/	Regulating macrophage polarization	/	[96]
circC3P1	MDSCs	Down-regulated	MiR-21	PTEN	Kidney cancer	Downregulating the protein levels of PTEN, restraining the PI3K/AKT and NF‐κB pathways	/	[72]
circSLC8A1	MDSCs	Down-regulated	MiR-130b/miR-494	PTEN, ARG1 and iNOS	Bladder cancer	Downregulating the protein levels of PTEN	/	[71]
circ-MTO1	MDSCs	Down-regulated	MiR-17-5p	ROS	Prostate cancer	Inhibiting the immunosuppressive function	/	[40]
cricFOREIGN	DCs	/	/	/	/	Activating DCs directly and indirectly activating CD4+ follicular T-helper (Tfh) cells and CD8+ T cells	/	[50]
f-circPR	Granulocytes	Up-regulated	/	/	Acute promyelocytic leukemia	Promoting tumorigenesis, tumor cell proliferation and cell transformation	/	[22]
f-circM9	Granulocytes	Up-regulated	/	/	Acute promyelocytic leukemia	Promoting tumorigenesis, tumor cell proliferation, cell transformation and resistance to arsenic trioxide	/	[22]
circ-HIPK2	Granulocytes	Up-regulated	MiR-124-3p	CEBPA	Acute promyelocytic leukemia	Affecting all-trans retinoic acid-induced differentiation of	/	[90]

#### CircRNA and T cells

CD8+ T cells can be activated by tumor-associated antigen (TAA) to specifically kill tumor cells. However, human tumors still progress as T cells invariably fail to eradicate the tumor owing to tumor-promoting molecular and cellular mechanisms, including T cell anergy, exhaustion, senescence and stemness [42]. CircRNAs participate in these immunosuppressive networks by impairing normal T cell function and enabling tumor escape.

CircRNAs regulate the T cell-mediated immune response, which is critical for tumor immunity. Correlation assays revealed that hsa_circ_0005519 activated the immune response, as it might induce cytokine IL-13 and IL-6 expression by regulating hsa-let-7a-5p in CD4+ T cells [43]. CircRNA hsa_circ_0012919 contributed to DNA methylation of CD70, a member of the TNF family, by sponging DNA methylation-related miRNAs in CD4+ T cells [44]. The CD27-CD70 interaction promotes the activation, proliferation and differentiation of T cells and B cells and plays an important role in mediating the immune response. Thus, circRNAs regulate specific cytokine secretion in CD4+ T cells, affecting the immune response against tumors.

Indoleamine 2,3-dioxygenase 1 (IDO1) suppressed the CD8+ T cell response in colon cancer, while miR-448, as a tumor-suppressive miRNA, enhanced the CD8+ T cell response by inhibiting IDO1 expression [45]. CircRNA circ-NT5C2 acted as an oncogene in tumor proliferation and metastasis by targeting miR-448 and subsequently decreased the immune response [46]. These data suggest that inhibition of circ-NT5C2 might strengthen the immune response against tumors. CircRNA-002178 could induce PD1 expression by sponging miR-34, which induces T-cell exhaustion [47].

Specific circRNAs mediate T cell aging, affecting immune senescence, one example is circRNA100783, which regulate phosphoprotein-related signal transduction during CD28-dependent CD8+ T cell aging [48]. Overlapping expression of circRNA100783 may represent a novel biomarker for the longitudinal tracking of CD28-related CD8+ T cell aging and global immune senescence [47]. Besides, circRNA in exosomes may participate in the regulation of Tregs [49].

CircRNAs are also notable regulators of T cell lymphoblastic lymphoma (T-LBL). Deng et al. found that circ-LAMP1, which was overexpressed in T-LBL tissues and cell lines, significantly boosted cell growth by inhibiting cell apoptosis in T-LBL cells. Circ-LAMP1 activated domain receptor tyrosine kinase 2 (DDR2) by sponging miR-615-5p, which directly targeted DDR2, a member of the receptor tyrosine kinase (RTK) family, therefore, circ-LAMP1 might be an oncogene in T-LBL, as RTK initiated a signaling cascade closely related to cancer progression. This finding may develop into a promising therapy for T-LBL [50].

As well, circRNAs are potent vaccine adjuvants that boost innate and adaptive immune responses. Study revealed that circRNAs transfected into HeLa cells stimulated innate immunity by enhancing the expression of specific genes which were highly related to response to cytokine, cytokine production, cellular response to virus, and NF-kB signaling [51]. Compared to endogenous circRNA with *N*6-methyladenosine(m^6^A) modification which could be recognized as “self” and wouldn’t stimulate immune response. Exogenous circRNAs without m^6^A modification bound to and activated RIG-1, which subsequently stimulated the activation of downstream signals and eventually increased the expression of immunity related genes, such as retinoic-acid-inducible gene-I (RIG-I), melanoma-differentiation-associated gene 5 (MDA5, also known as IFIH1), 2′-5′ oligoadenylate synthase 1 (OAS1) OAS-like protein (OASL) and protein kinase R (PKR). Comparative analysis of DCs isolated from C57BL/6 J mice which were injected cricFOREIGN and control subcutaneously revealed that cricFOREIGN could activate DCs and indirectly activated CD4 + follicular T-helper (Tfh) cells and CD8+ T cells by facilitating antigen cross-presentation. Also, OVA-B16 melanoma mice model vaccinated with circFOREIGN had significantly longer overall survival compared to negative control group [52]. Therefore, circRNAs can activate immunocytes to fight against tumors by acting as tumor antigens or by being modulated to enhance the immune response.

#### CircRNA and B cells

B cells, being a major cellular component in TME, are crucial effector cells in humoral immunity against tumor progression through secreting immunoglobulins, regulating tumor-suppressing responses of other immune cells directly and indirectly, such as T cells and macrophages [53]. Tertiary lymphoid structure (TLS), identified within a wide range of cancer tissues, are transient ectopic lymphoid aggregates with a similar structure and function of the secondary lymphoid organ [54]. Unique distribution, frequency of B cells and functional state of B cell-related pathways, including CCL19, -21/CCR7 axis and CXCL13/CXCR5 axis, take effect in enhancing immune response mainly via TLS formation [55]. A specific subset of infiltrating B cells are strong prognostic factors in various cancers context, such as CD20+ CD27− IgM+ group and CD20+ CD27− IgM− group in hepatocellular carcinoma(HCC), IgG4+ in pancreatic ductal adenocarcinoma and CD20+ in melanoma and some other cancers [56–59]. Several studies have also unveiled the significance of TLS formation in improving immunotherapy efficacy. Although the exact mechanisms have not been well understood, mediating B cell and B cell-related pathways are certainly vital to gain better cancer therapy outcome [58, 60, 61].

CircRNA can enhance the antibody response directly or indirectly [52]. Zheng et al. analyzed the circRNA expression profile of chickens inoculated with *Salmonella enterica* serovar enteritidis (SE). The results revealed that specific circRNA of which NFATC2 was the parental gene was related to B cell proliferation. CircRNA NC_006099.4:1 6132825|16236906 and circRNA NC_006099.4:15993284|16006290 mediated B cell proliferation through Foxp1 pathway [62]. Weng et al. compared and analyzed the expression profile of circRNAs between plasma of HCC patients with high tumor-infiltrating lymphocytes (TILs) and low TILs and identified that hsa_circ_0064428, which was significantly downregulated in HCC patients high TILs, was negatively correlated with patient prognosis [63]. Given the evidence above, hsa_circ_0064428 might be a key regulator of TIL formation with the potential to be utilized in B cell-related therapy.

#### CircRNA and natural killer cells (NKs)

NK cells constitute an early cellular defense mechanism that secretes cytokines and chemokines and employs cytotoxicity to reduce or damage pathogens or tumor cells. NK cells play an indispensable role in the immune system [64]. CircRNAs are notable regulators of the NK cell-mediated immune response. For example, hsa_circ_0008433 regulated inflammatory gene matrix metalloproteinases 2 (MMP2) expression by sponging hsa-miR-181c-5p and hsa-miR-181b-5p, inducing NK cells to attack arterial elastic fibers and remodel vessels, resulting in aneurysm progression [65, 66].

Tumor-induced circRNAs regulate NK cell activities. Androgen receptor (AR) differentially suppressed circRNA expression in HCC by upregulating adenosine to inosine acting on RNA enzyme 1 (ADAR1). ADAR1 directly suppressed RNA circularization, which had been observed for circARSP91 (hsa_circ_0085154). CircARSP91 enhanced innate immune surveillance by increasing the cytotoxicity of NK cells in HCC. As a repressor of HCC, enhancing circARSP91 activity was a potent novel therapy strategy [67].

Natural killer group 2 member D (NKG2D) on NK cells, LAK cells, and effector T cells mediate immune responses to cancer by interacting with different ligands on the tumor cell surface. Activation of the NKG2D ligand complex enhanced the immune response, leading to the subsequent lysis of tumor cells and thus prevented cancer progression [20]. A scatter plot analysis revealed a positive correlation between circTRIM33–12 expression and NKG2D-positive cell numbers in HCC tissues, indicating that circTRIM33–12 had a modulating effect on NKG2D. CircTRIM33–12 might exert its antitumor effects by enhancing the functions of NK cells [68].

Besides, the interaction of NKG2D with MHC class I-related molecule (MICA) was critical to the surveillance function of immune effectors in pancreatic cancer [69]. The interaction could be inhibited by NO via inhibition of hypoxia-inducible factor 1-alpha (HIF1A) accumulation [70]. Recently, Ou et al. found that circ_0000977 sponging miR-153, of which HIF1A was a downstream target, modulated HIF1A. Thus, overexpression of circ_0000977 promoted HI1FA accumulation, inhibiting NK cell lysis and resulting in immune escape of pancreatic cancer cells [71].

#### CircRNA and myeloid-derived suppressor cells (MDSCs)

MDSCs, derived from myeloid progenitor cells, comprise the major cell population that negatively regulates immune responses. Under pathological conditions, especially in tumors, MDSCs are aberrantly activated in the TME and release cytokines, such as reactive oxygen species (ROS), inducible NO synthase (iNOS), arginase 1 (ARG1) and other immunosuppressive cytokines, which all suppress the normal functions of T cells.

It has already been demonstrated that miR-494 in MDSCs is crucial to recruit MDSCs to the tumor site and regulate the production of ARG1 and iNOS by downregulating the protein levels of PTEN [72]. CircSLC8A1, generated from the SLC8A1 gene, directly interacted with miR-494, subsequently inhibiting the secretion of related cytokines [73]. CircRNA circC3P1 acted similarly by regulating the miR-21/PTEN axis [74].

Evidence suggested that miR-17-5p inhibited the expression of STAT3 and reduced the production of ROS, further inhibiting the immunosuppressive function of MDSCs [75]. Circ-MTO1 downregulated miR-17-5p expression in prostate cancer cells, which subsequently decreased ROS levels and inhibited cell proliferation and invasion [41]. The evidence above shows that in the TME, circRNAs regulate the fate of MDSCs; thus, circRNAs might serve as potential therapeutic targets by modulating the MDSC-mediated immune response.

#### CircRNA and granulocytes

Granulocytes are not only a crucial component of the innate immune response but also play pivotal roles in cancer progression, especially neutrophils which are the most abundant circulating leukocytes and a substantial proportion of the immune cell infiltrated in TME. Cancer-related neutrophils, including circulating neutrophils and tumor-associated neutrophils (TANs), can exert both pro-tumoral and antitumoral effects in different cancer context [76–78]. Circulating neutrophils serve as guards to escort circulating tumor cells which are precursors of cancer metastasis to travel in the bloodstream [79]. TANs can be polarized to antitumoral N1 phenotypes or pro-tumoral N2 phenotypes when exposed to different cues in TME. Pro-tumoral effects related evidence includes secreting pro-tumoral chemokines like myeloid growth factor granulocyte-colony stimulating factor (G-CSF) to promote cancer progression, suppressing T-cell mediated anti-tumor response and mediating degradation of IRS1 to boost cancer cell proliferation [80–84]. Anti-tumoral evidence includes presenting antigen, promoting T cell responses, putting down early tumor growth and resistance against primary 3-methylcholantrene-induced carcinogenesis [84–88]. A recent study directed by Ponzetta et al. found that neutrophils impelled the polarization of a subset of CD4- CD8- unconventional αβ T cells and type 1 immunity to fight against murine sarcomas and several human tumors [88]. In a word, the complex and intricate function of neutrophil in TME is vital to cancer progression which highlights its emerging role to be an effective therapeutic target.

A specific set of circRNAs are implicated in the normal function of granulocytes. In neutrophils, Toll-like receptor 6 (TLR6) generated a circRNA structure that functioned in the innate immune response [89]. Liang et al. demonstrated that circRNAs that were significantly decreased in severe acne compared with adjacent unaffected skin were involved in leukocyte trans endothelial migration [90]. The aberrant circRNA expression profile of the neutrophil transcriptome in patients with asymptomatic moyamoya disease (MMD) revealed that asymptomatic MMD was characterized by an intrinsic autoimmune status with different phenotypes of neutrophils, which had several differentially expressed circRNAs [91].

CircRNAs also play a crucial role in granulocyte-related cancer. PML/RARa is the most recurrent chromosomal translocation in patients with acute promyelocytic leukemia (APL). The fusion of the two translocated genes generates f-circRNAs. Guarnerio et al. mapped RNA-seq reads directly to linear RNA fusion and f-circRNA fusion reference libraries. A series of cell culture experiments revealed that f-circPR and f-circM9, together with other oncogenic hits, were biologically active, promoting tumorigenesis, tumor cell proliferation and cell transformation both in vitro and in vivo. F-circM9 also conferred tumor cell resistance to arsenic trioxide [22]. Li study also demonstrated that circ-HIPK2 served as a sponge for differentiation-associated miR-124-3p and significantly affected all-trans retinoic acid-induced differentiation of APL cells in APL patients, indicating its potential role as an APL biomarker [92].

#### CircRNA and macrophages

Macrophages are also effectors of innate immunity. They play crucial roles in linking innate and acquired immunity. Macrophages initiate innate immunity through special receptors called pattern‐recognition receptors, such as the Toll-like receptors (TLRs). After exposure to invading microorganisms or tumor cells, TLRs on the surface of macrophages recognize antigens or components of microorganisms, such as lipopolysaccharide (LPS), and initiate defense signaling cascades. During this process, cytokines and chemokines increase to enhance antigen presentation and other immune responses [93]. CircRNAs carry out specific functions in this pathway. Circ790 influenced the secretion of IL-6, IL-1β and TNF-α by macrophages [94]. Mouse circRasGEF1B (McircRasGEF1B), a kind of LPS-inducible circRNA that has a human homolog (hcircRasGEF1B) sharing similar properties, is a positive regulator of the LPS response. ICAM-1, an intercellular adhesion molecule involved in this response, facilitated the binding of leukocytes to endothelial cells and the subsequent transmigration into different tissues to promote the immune response. Knocking down the expression of mcircRasGEF1B with shRNA reduced LPS-induced ICAM-1 expression. Also, mcircRasGEF1B regulated the stability of mature ICAM-1 transcripts in LPS-activated cells [95].

In addition to preventing cytotoxic T cell (CTL) infiltration into the tumor core [96], TAMs are versatile cells that can rapidly polarize to accommodate different conditions. In response to microenvironmental stimuli, macrophages polarize to different phenotypes, including the M1 type activated by interferon-γ (IFN-γ) or other microbial components, such as LPS, and the M2 type activated by IL-4, IL-13 or the immune complex. CircRNA expression profiling revealed that M1 and M2 macrophages had different circRNA expression profiles, providing novel insight into the role of circRNAs in macrophage differentiation and polarization [31]. CircRNA-003780, circRNA-010056, and circRNA-010231 were upregulated in M1, with a fold change > 4 comparing with M2. Similarly, circRNA-003424, circRNA-013630, circRNA-001489 and circRNA-018127 were upregulated in M2 [68]. Another study revealed that circHECTD1 (HECT domain E3 ubiquitin-protein ligase 1) was involved in this polarizing process [97]. Additionally, ICAM-1 expression had been reported to suppress M2 macrophage polarization in the TME. Given the relationship between circRNAs and ICAM-1 discussed above, circRNAs are closely related to macrophage polarization [95].

### CircRNA and cancer-associated fibroblasts (CAFs)

CAFs are generated or differentiated from a subset of cells under cancer-bearing conditions, including local hypoxia and oxidative stress [98]. As the critical and most abundant component of the tumor mesenchyme, CAFs play key functions in the TME. CAFs provide physical support to other components, synthesize and modify the extracellular matrix, regulate other cell types in TME via bidirectional cell contact and release multiple regulatory factors to affect the occurrence and development of cancer in a context-dependent manner. They act as a double agent in tumors, as the complex and nuanced interactions between CAF cells and associated cells exert stimulatory (notably promoting metastasis) or inhibitory effects [99–102] depending on the spatial distribution of information [101] and surface markers. CAFs and fibroblasts share many prosperities, as tumors are “an unending series of wounds that continually initiate healing but never heal completely”. Understanding the relationship between circRNAs and fibroblasts is of substantial help to clarify the involvement of circRNAs in CAF regulation.

In primary human pulmonary fibroblasts (HPF-a) exposed to SiO, circHECTD1 was downregulated, thus inducing an increase in HECTD1, which subsequently caused fibroblast activation and accumulation via the EMT and endothelial-mesenchymal transition (EndMT) processes [97, 103, 104]. CircRNAs also help fibroblasts overcome stress. A study profiling differentially expressed circRNAs in the ultraviolet B stress-induced human fibroblast premature senescence (UVB-SIPS) model and assessing the role of circRNA_100797 in UVB-SIPS revealed that the decreased expression of circRNA_100797 had a photoprotective role in UVB-SIPS by sponging miR-23a-5p. The expression of circRNA_100797 in fibroblasts facilitated cell proliferation and alleviated cell cycle arrest [105]. Specific circRNAs disturbed fibroblast functions, such as circCOL3A1-859267, which was downregulated in UVA-exposed human dermal fibroblasts (HDFs). This circRNA regulated type I collagen expression by sponging miR-29c in human dermal fibroblasts [106].

There is sufficient evidence to classify that UV light are strong oncogenic factor of skin cancers, including melanoma, keratinocyte cancers, squamous cell carcinoma and basal cell carcinoma. UV radiation (both UV-A and UV-B) induces damage of skin cancer related genes, such as TP53, RAC1, and STK19 [107]. Fibroblasts in skin TME secrete more stimulating factors (basic fibroblast growth factor, hepatocyte growth factor and endothelin) after UV radiation exposure. Interactions between cancer cells and skin TME, including fibroblasts, promote cancer development of skin cancer cells [108, 109]. Although it is still unclear what role circRNA acts in the context of UV radiation leading skin cancer, circRNAs should play a part taken the evidence that circRNA regulates UV-exposed fibroblast above into consideration. CircRNA harnesses the potential to be utilized in treatment of skin cancer, especially developed from UV lesion.

Besides, circNFIB was found to be decreased in post-myocardial infarction mouse hearts and subsequently promoted adult fibroblast proliferation by sponging miR-433 [110]. Overexpression of circRNA_000203 could eliminate the anti-fibrotic effect of miR-26b-5p in cardiac fibroblasts [111]. Taken together, these results emphasize the importance of circRNAs to deregulate the functions of fibroblasts, indicating the potential importance of circRNAs in CAFs.

### CircRNA and ECM

ECM is composed of various macromolecules including fibronectin, collagens, proteoglycans and polysaccharides which are mainly secreted by CAFs. The bidirectional communication between ECM and cancer cell is crucial for cancer metastasis. Alterations in ECM, including composition and organization, is closed related to prognosis of cancer victims [112]. CircRNAs carried by exosomes disseminate from cancer cells to ECM and function as a regulator of ECM.

A functional enrichment analysis by Zou et al. revealed that circRNA CDR1 as, also named as ciRS-7, played a role in ECM reshaping, collagen binding and integrin binding. They also found CDR1as functioned as a regulator in ECM-receptor interaction, thereby mediating TME [113]. In cancer development, matrix metalloproteinases (MMPs) is vital for pathological destruction of ECM and malignant behavior of cancer cells. MMPs is upregulated in various tumors [114, 115]. Besides hsa_circ_0008433, hsa_circ_0000096 was also found to regulate MMP-2 and MMP-9 expression in gastric cancer. hsa_circ_0000096 levels was closed associated with several clinicopathological factors, including TNM stage, invasion and gender, presenting its clinical diagnostic value in gastric cancer [116]. These evidences suggest that circRNAs play a role in ECM remodeling, while the interplay and the exact mechanism needs further exploration.

### CircRNA and the vasculature

Angiogenesis is crucial to promote tumor growth and metastasis and has been identified as a hallmark of cancer. Tumor vasculature exhibits abnormal leaky structure and function compared to vessels in normal tissue and generates a special “hypoxia and malnutrition island” for cancer development. The island alters the expression of genes controlling the cancer stem cell compartment, epithelial-mesenchymal transition (EMT), and angiogenesis in tumor cells, hence promoting cell survival and resistance to apoptosis induction through a series of factors, such as HIF-1α [117] and ultimately influencing cancer development and therapeutic responses [118]. Hsa_circ_0014130 (circPIP5K1A), which is overexpressed in non-small cell lung cancer, facilitates cancer proliferation and metastasis by sponging miR-600, which interacts with the 3′ untranslated region of HIF-1α [119].

Despite the characterization of a series of ‘blood-vessel growth-stimulating factors’, vascular endothelial growth factor (VEGF) secreted by tumor cells and stroma in the TME is often considered to be a crucial angiogenic molecule in cancer [120]. The overexpression of VEGF in the majority, if not all, of human tumors, correlates strongly with poor outcomes in various cancers [121]. Several trials targeting the VEGF-VEGFR pathway to inhibit cancer have been conducted [122–124]. Vascular endothelial growth factor A (VEGFA) is a member of the VEGF growth factor family. Overexpression of VEGFA promotes angiogenesis, EMT and activates Ras/ERK signaling cascade, hence inducing tumor development [125]. The overexpression of circSCAF11 was found in glioma tissue specimens and cell lines and closely correlated with the poor clinical outcome of glioma patients. A study of circSCAF11 in glioma genesis demonstrated its molecular mechanism in the pathophysiological process. The upregulated molecule circSCAF11 sponged miR-421, thereby increasing SP1 expression and hence activating the transcription of VEGFA [126]. Circ0001429 and circRNA MYLK exerted similar functions in bladder cancer tissues via mediating VEGFA [125, 127].

VEGFA is not the only vehicle involved in the circRNA-angiogenesis regulating process. Belonging to the SOX gene family (Sex-related region Y, Sry-related high-mobility group box), the SOX13 gene had been proved to regulate angiogenesis in the human disease model [128]. He et al. found that circ_002136, overexpressed in glioma, functionally sponged miR-138-5p and subsequently enhanced SOX13 expression and regulated angiogenesis. The promoted SOX13 activated the upstream promoter FUS, forming a positive feedback loop to amplify its effect to regulate angiogenesis in glioma [129]. Another angiogenesis factor is forkhead box P1/P2 (FOXP1/FOXP2), which are targets of miR-544a/miR-379. FOXP1/FOXP2 are overexpressed since the upregulated molecule circ-SHKBP1 in glioma wound sequester their hunters-miR-544a/miR-379. Therefore, circ-SHKBP1 promoted the movement and tube formation of glioma-exposed endothelial cells to boost angiogenesis via the circ-SHKBP1/miR-544a/FOXP1 and circ-SHKBP1/miR-379/FOXP2 axis [130].

**Fig. 4:**
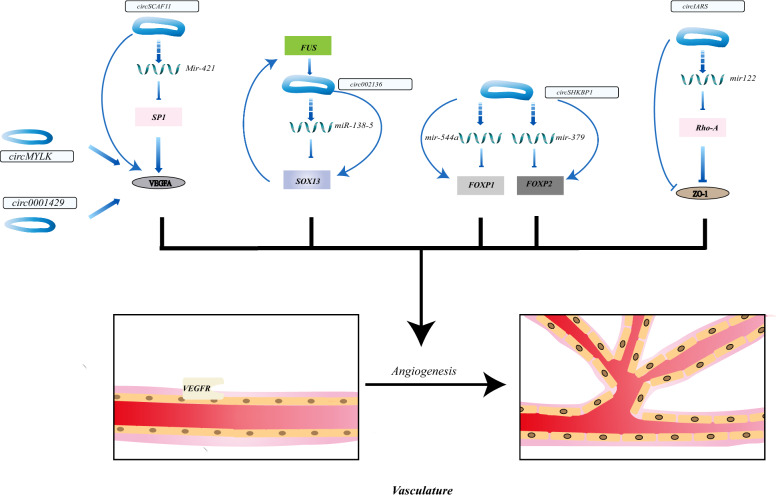
CircRNAs modulating angiogenesis in TME. Specific circRNAs act as promotor or inhibitor in angiogenesis. CircRNAs can increase the permeability of vessel wall

Apart from promoting angiogenesis, circRNA like circ-IARS which was secreted into exosomes by pancreatic cancer cells enhances the permeability of the vessel wall to accelerate cancer metastasis. The overexpressed circ-IARS sponged miR-122 and promoted the activity of Ras homolog gene family, member A (RhoA), which restrained tight junction ligand–protein Zonula occludens-1(ZO-1) and enhanced endothelial monolayer permeability, promoting cancer development [131]. These findings all give us a hint that targeting circRNAs to inhibit angiogenesis or rebuild the structure of vasculature is a promising approach to cancer therapy (Fig. 4, Table 2).

**Table 2:** Representative circular RNAs targeting angiogenesis

CircRNA	Expression	Targeted miRNA	Downstream target	Cancer type	Functions	Clinical correlation	Reference
circPIP5K1A	Up-Regulated	MiR-600	HIF-1α	Non-Small Cell Lung Cancer	Promoting angiogenesis by regulating VEGFR	/	[108]
circSCAF11	Up-Regulated	MiR-421	VEGFA	Glioma	Promoting angiogenesis by regulating VEGFR	Overall survival	[115]
Circ0001429	Up-Regulated	MiR-205-5P	VEGFA	Bladder Cancer	Promoting angiogenesis by regulating VEGFR	Overall survival	[116]
circular RNA MYLK	Up-Regulated	MiR-29A	VEGFA	Bladder Cancer	Promoting angiogenesis by regulating VEGFR	TNM stage, pathological grade	[114]
circ_002136	Up-Regulated	MiR-138-5P	SOX13	Glioma	Promoting angiogenesis by regulating SOX13	/	[118]
circ-SHKBP1	Up-Regulated	MiR-544A/Mir-379	FOXP1/FOXP2	Glioma	Promoting angiogenesis ability of endothelial cell by regulating FOXP1/FOXP2	/	[119]
circ-IARS	Up-Regulated	MiR-122	RhoA	Pancreatic Cancer	Increasing endothelial monolayer permeability to promote cancer development	Vascular invasion, TNM stage, liver metastasis, postoperative survival time	[120]

### CircRNA and vascular mimicry

Tumor cell vascular mimicry (VM), also known as vasculogenic mimicry, is a vessel like channel made up of tumor cells featuring cancer stem cell-like, trans-endothelial phenotype. Like real vessels, the de novo vascular structure also provides blood supply to nourish cancer cells and is closed associated to malignant behavior of various cancer and poor prognosis of cancer patients. Various molecular mechanisms are involved in VM, including MMP we talked in ECM section, VEGFR and HIF-1a in angiogenesis section, vascular endothelial (VE)-cadherin and phosphatidyl inositol 3-kinase (PI3K). Hypoxia is an important condition for VM, as it induces the functional plasticity of tumor cell and promotes VM. It is suggested that VM could be a potential therapeutic target to intensify angiogenic treatment [132–134].

Boeckel et al. found that circRNA ZNF292 (cZNF292) was upregulated under hypoxia circumstance and functioned as a promoter for angiogenesis in vitro, while the exact molecular mechanism remained unclear [135]. Yang et al. found that hypoxia-responsive manner of cZNF292 was independent of HIF1A and the knockdown cZNF292 in HCC SMMC7721 cells increased SRY (sex determining region Y)-box 9 (SOX9) nuclear translocation, which suppressed Wnt/β-catenin pathway and thereby inhibited cancer cell proliferation. Microscopic examination of vasculogenic mimicry density also revealed a decrease when cZNF292 was knocked down. The decrease of cancer cell proliferation should partially owe to the decrease of VM [136]. Similarly, Huang et al. found that exosomal circRNA-100,338 in serum of HCC patients could also regulate VM formation by upregulating VE-cadherin [137]. Their studies suggested circRNA played a part in VM regulation.

### CircRNA and tumor antigens

CircRNAs are generated specifically in tumorigenesis due to genetic mutations and chromosomal changes. Aberrant differentially expressed circRNAs may serve as tumor antigens to induce the immune response. Newly synthesized circRNAs in tumors may be packaged in exosomes [138] and transported to immunocytes [139] as tumor antigens to activate antitumor immunity or bind to miRNAs [140] and proteins to regulate immunocyte activity [51].

It remains incompletely clear how a foreign circRNA acting as a tumor antigen is sensed. The nucleic acid sensor RIG-I is a kind of pattern recognition receptor (PRR) for immune monitoring that can recognize 5′ triphosphate on short dsRNAs [141]. RIG-I, m^6^A modification and the immune factors NF90/NF110 are important regulators of the immune response and colocalize with foreign circRNAs [51, 52, 142]. Endogenous circRNAs with different introns that program their back-splicing will undergo m^6^A modification, suggesting that endogenous and exogenous circRNAs vary in their m^6^A modification. RIG-1 can bind both unmodified and m^6^A -modified circRNAs but can distinguish between them and can only be activated by the former. M^6^A is not the only RNA modification pathway involved in exogenous circRNA recognition [52, 143]. Once foreign circRNA is recognized, the double-stranded RNA-binding domain-containing immune factors NF90/NF110 promote circRNA biogenesis in the nucleus. They also interact with mature circRNAs in the cytoplasm to enhance the stability of circRNAs [142]. During viral infection, NF90/NF110 dissociate from circRNA-binding proteins and bind to viral mRNA to regulate antiviral immunity [91]. Chen et al. previously demonstrated that foreign circRNAs could trigger the immune response [51]. Given their stability and specificity, circRNAs can act as potent tumor antigens to enhance tumor immunity.

With bioinformatics analyses, circRNA databases such as MiOncoCirc [144] can be used to predict whether a circRNA can regulate tumor immunity-associated miRNAs, such as miR-148/152, miR-487b, and miR-17-92. Potential new circular tumor antigens may be identified based on these predictions.

## A perspective on circRNA in cancer therapy

With the rapid development of next-generation sequencing technology and bioinformatics tools, circRNAs are being increasingly identified. Extensive studies, numerous new software applications and databases have allowed us to gain detailed insight into the versatility of circRNAs. As multifaceted regulators, circRNAs contribute to tumor progression by modulating tumor cells directly or regulating the TME. In this review, we described the roles of circRNAs in tumor immunity, especially their roles in specific immune cell types.

Notably, the bidirectional communication between TME and specific cancer, such as pancreatic cancer, in which a small portion of cancer cell island immersed in the dense collagenous stroma, is tremendously crucial for the carcinogenesis, progression, and metastasis and therapy efficacy. Although the novel therapy such as programmed cell death-1 (PD-1) immune checkpoint inhibitor has made a great breakthrough, it has come to a dilemma as pancreatic cancer cells are endowed with the ability to escape or defend the therapy by the intangible, subtle and dynamic TME [145]. Hence, regulating TME by targeting the immune cells is substantial to break the bottleneck of immunotherapy for pancreatic cancer and some other cancers. Given the evidence above and existing related studies, the powerful regulating roles of circRNA in TME and their specific characteristics, such as stability and abundance, present circRNAs as promising targets to improve the treatment of pancreatic cancer.

CircRNAs can be utilized in tumor immunotherapy by serving as tumor antigens, vaccine adjuvants or working with other molecules, such as miRNAs or proteins, in immunocytes. Introducing circRNA which can suppress onco-miRNAs by serving as antisense into the targeted enemy is one approach as specific miRNAs are crucial regulators of carcinogenesis [146]. CircRNAs can be transferred into cells by various delivery techniques, such as exosomes or viroid. Artificially constructing and transporting specific circRNAs into target cells can affect the communication between normal cells and tumor cells in the carcinogenesis process, and some experiments have already shown promising results [147]. In osteosarcoma patients, circ-0000190 was downregulated in extracellular nanovesicles and tissues, which could be utilized to distinguish osteosarcoma patients. The encapsulated circ-0000190 delivered to osteosarcoma cells from normal cells impaired osteosarcoma cells’ ability to migrate, proliferate and invade, hinting that constructing artificial nanovesicles with circ-0000190 encapsulated could give osteosarcoma cells a strike [97].

However, the studies on circRNA are still in infant age, as the whole regulatory process is much more complicated. The regulating manner of circRNA could be tumor type-dependent and TME type-dependent. Algorithms and experiments detecting circRNA are not developed enough [38, 148]. Also, one controversy on circRNAs focuses on their expressive abundance. Physiological expressive levels of circRNAs may not be sufficient to sequester and suppress associated miRNAs. While most existing algorithms and experiments cannot take expressive levels into account, which makes some results not reliable. The relationship between circRNA and miRNA cannot be mapped to one-to-one, given that specific circRNAs interact with tens of miRNA, only if they have corresponding binding sites. The complex network involves many branches which may take positive or negative feedback roles in the downstream response. Besides, although the majority of circRNA do not have binding sites for miRNA, at present, most existing studies focus on the sponge function of circRNAs. The other functions, such as pseudogene translation and posttranscriptional regulation, have been underestimated. It is hard to crack these difficult questions. There is still a thick veil swathing on circRNAs. Therefore, the roles of circRNAs in the TME are still a gold mine waiting to be explored further.

## Conclusions

Reprogramming TME is a potent strategy to eradicate tumors, and new targets need to be characterized. CircRNAs are multifunctional molecules that play essential roles in tumor progression. CircRNAs inducing aberrant functions in the TME can be valuable new targets to treat cancer or become novel biomarkers for immunotherapy. In this review, we discussed the crosstalk of circRNAs between immune cells, CAFs, and the vasculature in the TME and summarized the potential clinical applications of circRNA-based therapeutics. Considering the evidence collectively, we believe that circRNA-based therapeutics will contribute promisingly to treat cancer.

## Data Availability

Data sharing is not applicable to this article as no datasets were generated or analyzed during the current study.
